# A Table of All the Known Operations of Ovarotomy from 1701 to 1851

**Published:** 1852-07

**Authors:** 


### EDITORIAL DEPARTMENT.

---

*A Table of all the known Operations of Ovariotomy from* 1701 *to* 1851, *comprising two hundred and twenty-two cases; including their Synoptical History and Analysis.* By Washington L. Atlee, M. D., Prof. of Medical Chemistry in the Med. Dept. of Pennsylvania College Philadelphia.

This truly valuable statistical paper is contained in the Transactions of the Am. Med. Association, but has been issued separately for private distribution. The title-page sufficiently sets forth the character of the work, which, together with the numerous operations performed by Dr. Atlee, will always associate his name with the literature of the subject.

In an appendix to the table, Dr. Atlee presents certain facts which, in justice to his labors, should be generally known. It appears that the successful competitor for a prize on this subject, in London, Eng., Dr. Thomas S. Lee, appropriated from the Am. Journal of Med. Sciences the table of Dr. Atlee, published in that periodical in 1845, without the least acknowledgment. He uses, not only the facts collected by Dr. Atlee, but his arrangement, and even his very language, without giving any intimation that they were borrowed. Dr. Atlee, feeling justly aggrieved, addressed a note to Dr. Lee, appealing to his sense of honor, and calling attention to the omission of reference to the source whence all that was truly valuable in his memoir was derived. Dr. Lee acknowledged the injustice, and promised to make reparation in another edition.

The plagiarism of Dr. Lee hardly admits of any apology, but it seems that the injustice to Dr. Atlee was not limited to the British Author. Dr. Meigs, of Philadelphia, in his work entitled *Females and their Diseases*, etc., quotes in full the table from Dr. Lee's memoir, giving the whole credit to Dr. L., designating it "Dr. Lee's table." In so doing Dr. Meigs, it may charitably be supposed, was deceived, as were doubtless those who awarded the prize to Dr. Lee, and other readers. But in the second edition of his treatise on females, after his attention had been called to the subject, Dr. Meigs sees fit to omit altogether Dr. Lee's tables, as he styles them, but still alludes to them as belonging to Dr. Lee, making no mention of the name of Dr. Atlee in connection therewith.

These facts should be known. The reader will, of course, form his own conclusions respecting them.

---

*Fever Report.*—Perhaps the Editor should apologize for the space in this No. occupied by the concluding portion of his Third Report on Continued Fever. A desire not to extend the subject over several numbers, led to the insertion of the whole of the remainder of the Report in the present issue. In the issue for August he proposes to contribute two articles relating to Continued Fever. One will embrace a digest of the morbid appearances after death, exclusive of the intestinal lesions, from papers by Dr. Wm. Jenner, of London. The other will relate to "*Relapsing Fever*," including an analysis of the cases, contained in the Second Report, characterized by relapses. After the publication of these two articles, the subject will be dropped for other *novelties*.

